# The Effect of Static and Dynamic Stretching Exercises on Sprint Ability of Recreational Male Volleyball Players

**DOI:** 10.3390/ijerph16162835

**Published:** 2019-08-08

**Authors:** Foteini Alipasali, Sophia D. Papadopoulou, Ioannis Gissis, Georgios Komsis, Stergios Komsis, Angelos Kyranoudis, Beat Knechtle, Pantelis T. Nikolaidis

**Affiliations:** 1 Department of Physical Education & Sport Science, Aristotle University of Thessaloniki, 62100 Serres, Greece; 2 Laboratory of Evaluation of Human Biological Performance, Department of Physical Education & Sport Science, Aristotle University of Thessaloniki, 57001 Thessaloniki, Greece; 3 Department of Physical Education & Sport Science, Democritus University of Thrace, 69100 Komotini, Greece; 4 Institute of Primary Care, University of Zurich, 8091 Zurich, Switzerland; 5 Exercise Physiology Laboratory, 18450 Nikaia, Greece

**Keywords:** dynamic stretching, static stretching, velocity, volleyball, warm-up

## Abstract

The aim of the present trial was to investigate the effect of two stretching programs, a dynamic and a static one, on the sprint ability of recreational volleyball players. The sample consisted of 27 male recreational volleyball players (age 21.6 ± 2.1 years, mean ± standard deviation, body mass 80.3 ± 8.9 kg, height 1.82 ± 0.06 m, body mass index 24.3 ± 2.5 kg.m^−2^, volleyball experience 7.7 ± 2.9 years). Participants were randomly divided into three groups: (a) the first performing dynamic stretching exercises three times per week, (b) the second following a static stretching protocol on the same frequency, and (c) the third being the control group, abstaining from any stretching protocol. The duration of the stretching exercise intervention period was 6 weeks, with all groups performing baseline and final field sprinting tests at 4.5 and 9 m. The post-test sprint times were faster in both the 4.5 (*p* = 0.027, η^2^ = 0.188) and 9 m tests (*p* < 0.001, η^2^ = 0.605) compared to the pre-test values. A large time × group interaction was shown in both the 4.5 (*p* = 0.007, η^2^ = 0.341) and 9 m tests (*p* = 0.004, η^2^ = 0.363) with the static and dynamic stretching groups being faster in the post-test than in the pre-test, whereas no change was found in the control group. The percentage change in the 4.5 m sprint time correlated with volleyball experience (*r* = −0.38, *p* = 0.050), i.e., the longer the volleyball experience, the larger the improvement in the 4.5 m sprint. Thus, it is concluded that both stretching techniques have a positive effect on the velocity of recreational male volleyball players, when performed at a frequency of three times per week for 6 weeks under the same conditions as defined in the study protocol.

## 1. Introduction

Today, the main goal of athletic training and sports participation is to ameliorate performance. Performance however, is multifactorial, depending on several parameters, including warm-up practices. The purpose of warming up is to prepare the athlete for the upcoming sports event in a physiological view point, making the transition from the resting state to the state of preparedness needed for sports competition [1,2]. It is common for stretching exercises to be performed between the general and specialized parts of the warm-up session, with dynamic stretching being more preferred lately as opposed to static stretching. Stretching exercises are considered a pivotal effector of joint flexibility [2,3,4], adding biomechanical precision to an athlete’s movement while offering the opportunity to perform at maximum force throughout the range of motion [5,6].

Although the literature provides ample evidence on the acute effects of static and dynamic stretching exercises on performance [1,2,7,8], the number of studies on the chronic effects of both static [9,10,11] and dynamic stretching are limited and appear inconclusive [12,13,14,15]. Passive stretching is associated with an eccentric elongation of the muscle [16], while on the other hand, energetic stretching induces concentric elongation with parallel increments in the muscle perimeter. It is hypothesized that new sarcomeres are formed in line during passive stretching [17,18], whereas when adhering to a dynamic stretching protocol new muscle fibers are produced, with a parallel sarcomere formation. It should be noted, however, that flexibility improvements associated with muscle elongation have an additional effect on muscle performance [19].

Volleyball is one of the sports where stretching is usually incorporated in the warming up procedure. During a volleyball match, the high vertical jump and the explosive movements performed to cover court space are considered of utmost importance, and are highly intercorrelated [20]. During the match, a volleyball player tends to cover distances ranging between 4.5 and 9 m [21], and due to these small distances as compared to other sports, sprint and acceleration are pivotal acquisitions of a successful volleyball player. Additionally, only few seconds or milliseconds are required when moving towards the ball, and this is why accurate sprint measurements are performed, using photocells with a precision of milliseconds [22].

Sprints are important components of team sports, with the majority of research reporting reductions in speed immediately after the performance of static stretching exercises [23,24,25]. Nevertheless, research examining the sprinting ability of athletes after a long-term adherence to static stretching protocols has been limited and has provided conflicting findings [9,12,26]. According to the research, no differences were observed in the sprinting ability with agility changes after the implementation of either a 4 week [12] or a 6 week [9] lower-limb static stretching protocol, whereas the 20 m sprint time was significantly improved after performing static stretching exercises for a total of 10 weeks [26].

On the other hand, as far as dynamic stretching is concerned, it is reported to acutely improve the sprint time [23,27]. Research assessing sprinting ability post the implementation of dynamic stretching protocols lasting for a few weeks is limited, providing controversial results [12,14]. For example, when a 4 week lower-limb dynamic stretching program was followed, improvements in the sprinting ability with agility changes have been reported by some [12], whereas others [14] failed to record differences in the sprinting ability after an 8 week protocol. Given the controversial literature findings, the aim of the present trial was to investigate the effect of two stretching programs, a dynamic and a static one, on the sprint ability of recreational volleyball players.

## 2. Materials and Methods

### 2.1. Participants

A total of 50 male, apparently healthy physical education undergraduate students, all recreational volleyball players, participated in the study. The participants were randomly assigned into three groups (static, *n* = 17; dynamic, *n* = 17; control group, *n* = 16). The term “recreational” denotes that participants were volleyball players of teams competing at the regional level. Two participants were excluded due to injury during the course of the trial and six were excluded for not completing the trial, leaving a total sample of 42 participants. Among them, complete data of demographic characteristics (age, body mass, height, volleyball experience) and sprint ability (4.5 and 9 m sprint times) were available for 27 participants (Table 1), who were included in the present analysis. Participants volunteered for study participation during the volleyball module offered by the Aristotle University of Thessaloniki. Their volleyball experience was defined as the years they had been practicing volleyball as members of sport clubs that involved three to four training units during weekdays and an official match during the weekend. All participants were informed of the exact nature, procedures, and aim of the trial before providing informed consent to participate. Ethical permission was granted from the Aristotle University’s Ethics Committee and all procedures were in accordance with the Declaration of Helsinki for research on human subjects.

### 2.2. Design and Procedures

The study was conducted from the middle of February 2015 until the end of March 2015. Both testing and stretching exercise sessions were performed in the indoor court of the School of Physical Education and Sport Sciences of Aristotle University of Thessaloniki. All stretching exercise sessions of both the static and dynamic groups were supervised by the principal investigator of this study (F.A.) and were administered individually, i.e., one-by-one. During the 6 week period of the study, participants were strictly instructed to maintain their regular physical activity and nutritional habits. Participants were randomized into three groups, each following a different protocol, with every protocol lasting for a total of 6 weeks as, according to the literature, this is the minimum time required to produce effective changes in the joint range of motion (ROM) [13]. The baseline characteristics of participants were presented in Table 1. The first group adhered to a static stretching protocol performed three times per week, the second followed a dynamic stretching protocol performed in the same frequency, and the last one abstained from any stretching exercises for the duration of the trial, forming the control group. During the trial, all participants continued to follow their everyday activities, but additionally incorporated the protocol of the group in which they were placed for the duration of the trial. All three groups participated in baseline and post-protocol 4.5 and 9 m sprint tests.

**Figure 1:**
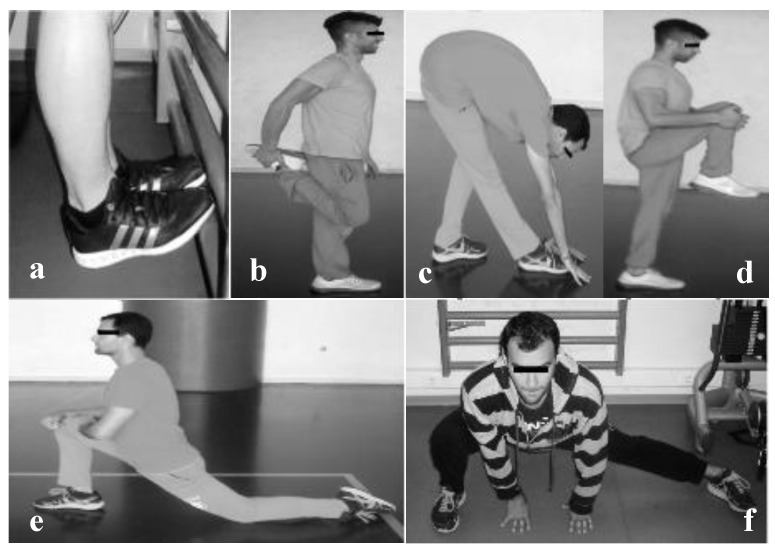
Static stretching protocol exercises of the (**a**) posterior tibial, (**b**) front crural, (**c**) posterior crural, (**d**) gluteus, (**e**) iliopsoas, and (**f**) topside muscles.

The static stretching protocol included static stretching exercises of the lower limbs (posterior tibial muscles, front and posterior crural muscles, topside and iliopsoas muscles), for a total duration of 4 min. Each stretching exercise lasted for 10 s and was repeated twice (2 × 10 s), with a 10 s break between exercises using both limbs simultaneously and without any break for exercises using one limb at a time. All exercises were performed in the maximum joint ROM, while avoiding muscle pain (Figure 1).

**Figure 2:**
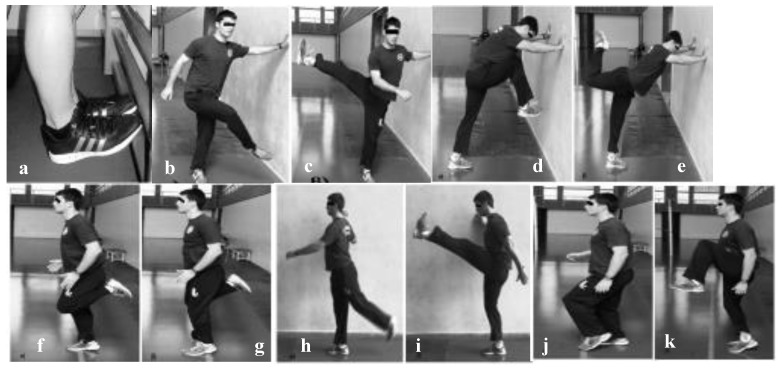
Dynamic stretching protocol exercises of the (**a**) posterior tibial, (**b**,**c**) topside, (**d**,**e**) iliopsoas, (**f**,**g**) front and (**h**,**i**) posterior crural, and (**j**,**k**) gluteal muscles.

The second protocol involved 6 weeks of dynamic stretching exercises, implemented in the same frequency as the first one (three times per week). It involved dynamic stretching exercises performed in the exact same manner as the first protocol (Figure 2). Finally, the third protocol (control protocol) involved abstaining from stretching exercises for the total duration of the trial (6 weeks). 

**Figure 3:**
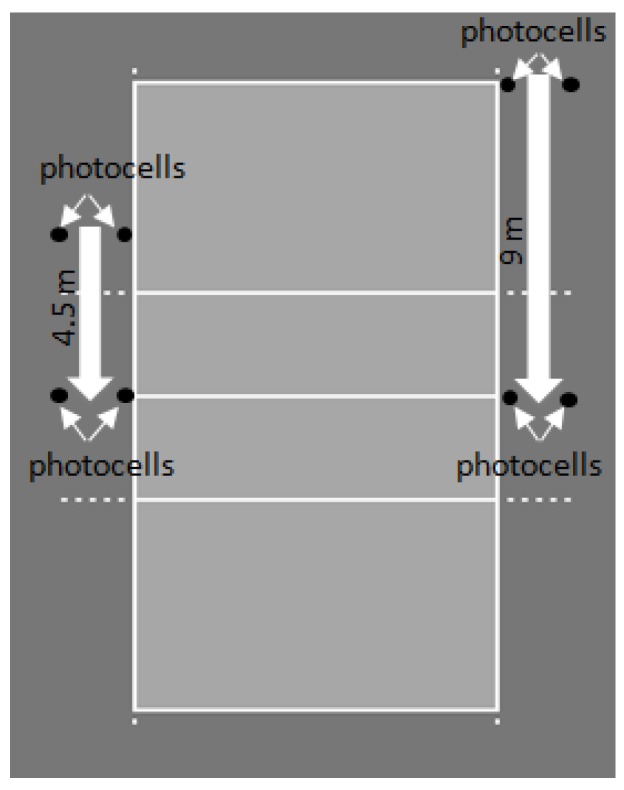
Sprint tests procedure.

The sprint tests were performed inside the volleyball court, in line with the parallel end of the court. Two maximal sprint tests were carried out at 4.5 m, and the one with the best result was kept for each participant (Figure 3). Initially, participants warmed up by performing submaximal intensity sprints towards different directions, including side movements, for a total duration of 5 minutes without any stretching exercises. Then, sprint tests were carried out on the side of the court (Figure 3). Participants were asked to start the sprint in random order, with their body in standing position and their knees slightly bent, with one leg (right or left) approximately 40 cm behind the starting line, entering from the beam gate where the first pair of photocells was placed. Then they ran towards the finishing line where the second pair of photocells was placed. Instructions were provided on running as fast as possible, without slowing down towards the finishing line. Each participant initiated the trial alone, without receiving any signal from the examiners.

The same procedure was followed for the 9 m sprint test, which was also performed on the side of the court. A break lasting for more than 3 min intervened between each sprint [28]. The running speed was measured using the two pairs of photocell shutters and a digital chronometer [28]. Τhe velocity assessment was carried out with a dual-beam photocell system (Autonics Beam Sensor BL5M-MFR) and a digital timer (Saint Wien Digital Timer Type H5K).

### 2.3. Statistical Analysis

Statistical analysis was carried out using SPSS software (IBM, New York, NY, United States of America) and the level of significance was set at α = 0.05. Between- and within-subjects analyses of variance examined the main effects of group (static, dynamic, and control), time (pre- and post-test), and group × time interaction on sprint times of 4.5 and 9 m. A post hoc Bonferroni test examined differences among the static, dynamic, and control groups. The percentage difference (Δ%) in sprint time from pre- to post-test was calculated using the formula ‘100 × (sprint time at post-test − sprint time at pre-test)/sprint time at pre-test’. The relationship of Δ% in sprint time with demographic characteristics was examined using Pearson correlation coefficient *r*, whose magnitude was interpreted as trivial (*r* < 0.10), small (0.10 ≤ *r* < 0.30), moderate (0.30 ≤ *r* < 0.50), large (0.50 ≤ *r* < 0.70), very large (0.70 ≤ *r* < 0.90), nearly perfect (*r* ≥ 0.90), or perfect (*r* = 1.00) [29].

## 3. Results

**Figure 4:**
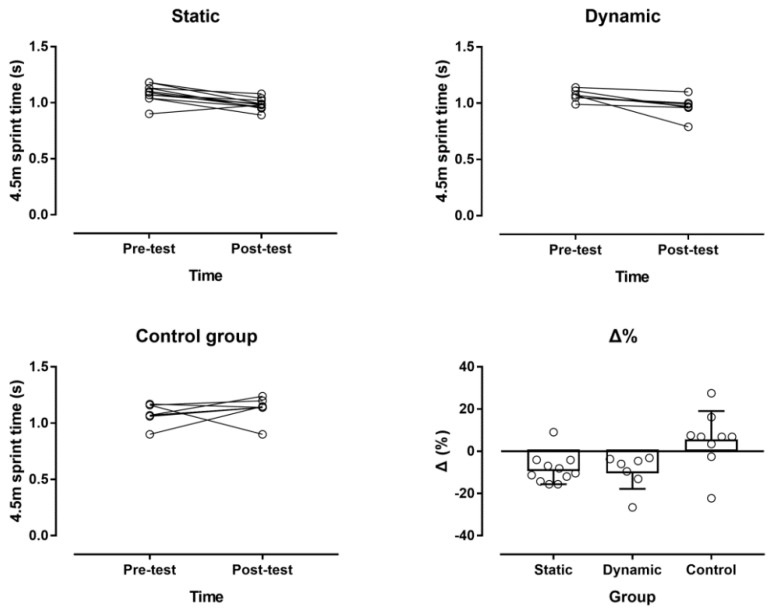
Individual changes in the 4.5 m sprint time by experimental group and percentage change (Δ%).

In the 4.5 m sprint time, a large main effect of time was observed (*p* = 0.027, η^2^ = 0.188), where overall the post-test was faster than the pre-test sprint time (1.03 ± 0.11 s and 1.08 ± 0.07 s, respectively; mean difference −0.05 s; 95% confidence intervals, CI, −0.09, −0.01) (Figure 4). A large time × group interaction was shown (*p* = 0.007, η^2^ = 0.341), with the static and dynamic stretching groups being faster in the post-test than in the pre-test, whereas no change was found in the control group. Overall, the static and dynamic stretching groups were faster than the control group by −0.07 s (95% CI, −0.13, −0.01) and −0.09 s (95% CI, −0.16, −0.02), respectively.

**Figure 5:**
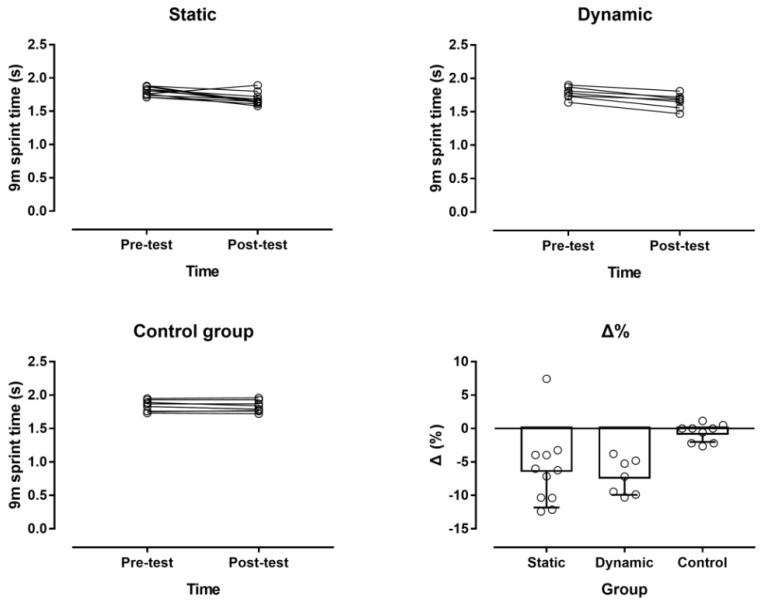
Individual changes in the 9 m sprint time by experimental group and percentage change (Δ%).

In the 9 m sprint time, a large main effect of time was observed (*p* < 0.001, η^2^ = 0.605), where overall the post-test was faster than the pre-test sprint time (1.72 ± 0.12 s and 1.81 ± 0.08 s, respectively; mean difference −0.08 s; 95% CI, −0.11, −0.06) (Figure 5). A large time × group interaction was shown (*p* = 0.004, η^2^ = 0.363), with the static and dynamic stretching groups being faster in the post-test than in the pre-test, whereas no change was found in the control group. Overall, the static and dynamic stretching groups were faster than the control group by −0.09 s (95% CI, −0.18, 0) and −0.11 s (95% CI, −0.21, −0.01), respectively.

**Figure 6:**
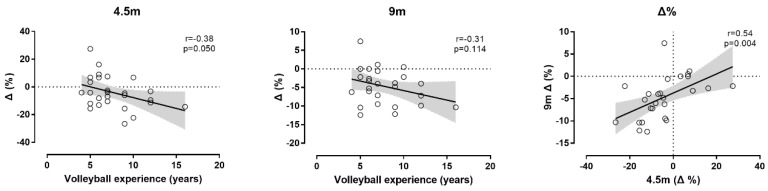
Relationship of percentage change (Δ%) from pre-test to post-test between sprint ability and volleyball experience.

With regard to the relationship of changes in the sprint ability from pre- to post-test with demographic characteristics of participants, a moderate negative correlation of percentage change in the 4.5 m sprint with volleyball experience was observed; i.e., the longer the volleyball experience, the larger the improvement in the 4.5 m sprint (Figure 6). The percentage change in the 4.5 m sprint correlated largely with the percentage change in the 9 m sprint. No relationship was observed in the relationship of age, weight, height, or body mass index with percentage changes in sprint ability (*p* > 0.05).

## 4. Discussion

The present study examined the effects of 6 week static and dynamic stretching exercise protocols on the sprint speed of recreational volleyball players. The main finding of the study was that the time to complete the 4.5 and 9 m sprint tests significantly improved after the implementation of dynamic and static stretching protocols. A secondary finding was that both 4.5 and 9 m sprint tests had similar sensitivity to evaluate chronic adaptations to stretching exercise programs.

Similar findings have been reported among wrestlers performing dynamic stretching five times per week for a total of 4 weeks [12]. Adherence to long-term dynamic stretching appears to improve sprinting time as a result of dynamic muscle elongation and coordination improvement [30], reducing energy cost [31] while paving the way for re-usage of the elastic strain energy [32]. Time to complete the 4.5 and 9 m sprint tests was also improved in the static stretching protocol team. Similar improvements were reported by Kokkonen et al. [26] on men and women performing static stretching three times per week for a total of 10 weeks. Bazett-Jones et al. [9], on the other hand, failed to record any improvements in sprinting ability 6 weeks after a static stretching warm-up scheme, performed at a frequency of four times per week. Their sample included female athletes of classic sports; however, it is well known that women are less affected by static stretching due to the already high flexibility they attain as a result of the inner gastrocnemius muscle tendon [33]. According to Earp et al. [34], muscle contraction speed and the ability to perform power exercises are both improved in line with muscle fiber elongation. Thus, the improvement in the sprinting tests herein could be attributed to an improvement in muscle fiber length. On the other hand, the control group failed to demonstrate any improvements. This was expected, given that participants of this group did not adhere to any exercise/warm-up protocol affecting muscle elongation during the 6 week trial.

In addition, it should be highlighted that both tests (4.5 and 9 m) indicated improvement of sprint ability at 6 weeks of dynamic and static stretching protocols. This similarity between these two sprint tests suggested their physiological affinity. Previous research in soccer showed that sprint tests—e.g., 10 versus 20 m—are related to similar anthropometric and physiological characteristics [35,36]. For instance, both the 10 and 20 m sprints correlated positively with body mass and height, and negatively with squat jump, countermovement jump, and peak power in the Wingate anaerobic test [35]. With regard to the relationship of change in the 4.5 m sprint time from pre- to post-test with volleyball experience, the larger improvements in sprint time observed in the more experienced participants compared to their less experienced counterparts highlighted the relationship between trainability and volleyball experience.

A limitation of the present study was that it used a specific set of either dynamic or static stretching exercises; thus, the findings should be generalized with caution to stretching exercise programs consisting of different stretching exercises or exercise characteristics (e.g., exercise intensity, volume, and frequency). Moreover, further research could examine—using larger sample sizes—the relationship of longitudinal changes in sprinting ability and anthropometric characteristics, as well as the role of nutrition, since it has been shown that physical performances in volleyball are related to anthropometric characteristics [37]. On the other hand, the strength of the study was its novelty considering the relatively small number of previous research works on the chronic adaptations of sprint ability to dynamic and static stretching exercise. Since stretching exercises are a major component of exercise programs, knowledge of their impact would be of great practical importance for professionals (e.g., physicians, sport scientists) who prescribe exercise. 

## 5. Conclusions

The present study shows that both static and dynamic stretching protocols have a positive effect on sprinting time when implemented for a total of 6 weeks, three times per week. Additionally, the protocols used herein could be of use to trainers for systematic implementation among athletes of different sports, including volleyball, in an effort to improve sprint ability.

## Figures and Tables

**Table 1:** Demographic characteristics of participants in the experimental group.

Variable	Total (*n* = 27)	Static Group (*n* = 11)	Dynamic Group (*n* = 7)	Control Group (*n* = 9)
Age (years)	21.6 ± 2.1	21.4 ± 2.0	22.4 ± 2.1	21.3 ± 2.3
Weight (kg)	80.3 ± 8.9	76.5 ± 7.9	84.5 ± 10.4	81.7 ± 8.0
Height (m)	1.82 ± 0.06	1.79 ± 0.04	1.85 ± 0.07	1.83 ± 0.05
BMI (kg.m^-2^)	24.3 ± 2.5	24.0 ± 2.6	24.6 ± 1.9	24.6 ± 3.1
Volleyball experience (years)	7.7 ± 2.9	7.5 ± 3.6	9.1 ± 2.3	6.8 ± 2.0

BMI = body mass index.
